# Spectrophotometric Determination of Gemifloxacin Mesylate, Moxifloxacin Hydrochloride, and Enrofloxacin in Pharmaceutical Formulations Using Acid Dyes

**DOI:** 10.1155/2014/286379

**Published:** 2014-01-22

**Authors:** Ayman A. Gouda, Alaa S. Amin, Ragaa El-Sheikh, Amira G. Yousef

**Affiliations:** ^1^Chemistry Department, Faculty of Science, Zagazig University, Zagazig 44519, Egypt; ^2^Chemistry Department, Faculty of Science, Benha University, Benha, Egypt

## Abstract

Simple, rapid, and extractive spectrophotometric methods were developed for the determination of some fluoroquinolones antibiotics: gemifloxacin mesylate (GMF), moxifloxacin hydrochloride (MXF), and enrofloxacin (ENF) in pure forms and pharmaceutical formulations. These methods are based on the formation of ion-pair complexes between the basic drugs and acid dyes, namely, bromocresol green (BCG), bromocresol purple (BCP), bromophenol blue (BPB), bromothymol blue (BTB), and methyl orange (MO) in acidic buffer solutions. The formed complexes were extracted with chloroform and measured at 420, 408, 416, 415, and 422 nm for BCG, BCP, BPB, BTB, and MO, respectively, for GMF; at 410, 415, 416, and 420 nm for BCP, BTB, BPB, and MO, respectively, for MXF; and at 419 and 414 nm for BCG and BTB, respectively, in case of ENF. The analytical parameters and their effects are investigated. Beer's law was obeyed in the ranges 1.0–30, 1.0–20, and 2.0–24 **μ**g mL^−1^ for GMF, MXF, and ENF, respectively. The proposed methods have been applied successfully for the analysis of the studied drugs in pure forms and pharmaceutical formulations. Statistical comparison of the results with the reference methods showed excellent agreement and indicated no significant difference in accuracy and precision.

## 1. Introduction

**Scheme 1:**
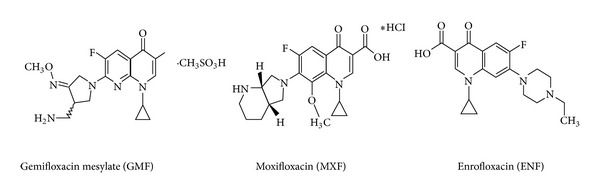
The chemical structure of the studied fluoroquinolones.

Fluoroquinolones are the second-generation members of quinolone antibiotics fluorinated in position 6 and bearing a piperazinyl moiety at position. They are considered to be the most effective Gram-positive and Gram-negative pathogens to combat infection caused by microorganisms that are resistant to other microbials, such as tetracyclines. Also, they have some activity against mycobacteria, mycoplasmas, rickettsias, and the protozoan *Plasmodium falciparum* [1–3]. There is a substantial body of literature related to both the mechanism of their action as DNA gyrase inhibitors and the influence of systematic structural modifications on their biological activity. Gemifloxacin mesylate (GMF) is (R,S)-7-[(4Z)-3-(aminomethyl)-4-(methoxyimino)-1-pyrrolidinyl]-1-cyclopropyl-6-fluoro-1,4-dihydro-4-oxo-1,8-naphthyridine-3-carboxylic acid methanesulfonate. Moxifloxacin (MXF) is {1-cyclopropyl-7-[2,8-diazobicyclo (4.3.0) nonane]-6-fluoro-8-methoxy-1,4 dihydro-4-oxo-3-quinolone carboxylic acid}. Enrofloxacin (ENF) is (1 cyclopropyl-7-(4-ethyl-1-piperazinyl)-6-fluoro-1,4-dihydro-4-oxo-3-quinolone carboxylic acid) (Scheme 1). GMF and MXF are fourth-generation synthetic broad-spectrum 8-methoxy fluoroquinolone antibacterial drug derivatives. Due to their clinical advantages, GMF and MXF are receiving a great interest and there was an increase in number of their pharmaceutical dosage forms in the market in the recent past. Enrofloxacin is the first fluoroquinolone developed for veterinary application and is potentially available for the treatment of some urinary tract, respiratory tract, and skin infectious diseases in pets and livestock [2]. There are no official (pharmacopoeia) methods that have been found for the assay of GMF and MXF in their pharmaceutical formulations. ENF is official in the United States Pharmacopeia (USP) [4].

Several methods have been reported for the determination of fluoroquinolones either in pure forms, dosage forms, or biological fluids like chromatography [5–9], microchip electrophoresis [10], chiral counter-current chromatography [11], capillary zone electrophoresis [12], electrochemistry [13–15], atomic absorption spectrometry [16, 17], and spectrofluorimetry [18–21]. However, these methods are expensive and not available at most quality control laboratories. For routine analysis of the studied drugs, a simple, rapid, and cost effective analytical method was required.

The spectrophotometric technique continues to be the most preferred method for the assay of different classes of drugs in pure, pharmaceutical formulations and in biological samples, for its simplicity and reasonable sensitivity with significant economical advantages. Spectrophotometric methods are reported for the assay of GMF [22–32], MXF [16, 33–40], and ENF [17, 41–47]. These methods were associated with some major drawbacks such as decreased selectivity due to measurement in ultraviolet region and/or decreased simplicity of the assay procedure (e.g., tedious precipitation, heating, or liquid-liquid extraction steps in the ion-pair formation-based methods). For these reasons, it was worthwhile to develop a new simple and selective spectrophotometric method for the determination of the studied drugs in their pharmaceutical dosage forms.

In the present work, we report the development of accurate and precise extractive spectrophotometric methods based on the chloroform soluble ion-pair complexes between the studied fluoroquinolone antibiotics (GMF, MXF, and ENF) and some acid dyes (BCG, BCP, BTB, BPB, or MO). The absorbance measurements were measured at optimum wavelengths. The proposed methods were applied successfully for the determination of the studied drugs in pure and dosage forms. No interference was observed from the additives. The methods provide rapid, economic procedures and more sensitive compared to the previously reported spectrophotometric methods. These methods were validated by the statistical data.

## 2. Experimental

### 2.1. Apparatus

All absorption spectra were made using Kontron Unikon 930 (UV-Visible) spectrophotometer (German) with a scanning speed of 200 nm/min and a band width of 2.0 nm, equipped with 10 mm matched quartz cells. The pH values of different buffer solutions were checked using a Hanna pH-meter instrument (pH 211) (Romania) equipped with a combined glass-calomel electrode.

### 2.2. Materials and Reagents

All reagents and chemicals used were of analytical or pharmaceutical grade and all solutions were prepared fresh daily.

*Materials.* Pharmaceutical grade gemifloxacin mesylate (GMF) was supplied by Al-Obour Pharmaceutical and Chemical Industries Company, Egypt. Moxifloxacin hydrochloride (MXF) reference standard was provided by Sabaa, Kahira Company, Egypt. Enrofloxacin (ENF) was kindly provided by Pharma Swede, Egypt (AVICO).

All pharmaceutical preparations were obtained from commercial sources in the local markets. Factive tablets were obtained from Oscient Pharmaceuticals Corporation, USA, labeled to contain 320 mg GMF per tablet; Flobiotic tablets were obtained from Hikma Pharmaceutical and Chemical Industries Company, Egypt, labeled to contain 320 mg GMF per tablet. GemiQue tablets were obtained from Obour Pharmaceutical and Chemical Industries Company, Egypt, labeled to contain 320 mg GMF per tablet. Avelox tablets were obtained from Bayer, Germany, labeled to contain 400 mg MXF per tablet. Moxiflox tablets were obtained from EVA Pharm. & Chem. Ind. Company, Egypt, labeled to contain 400 mg MXF per tablet. Moxifloxacin tablets were obtained from Sabaa International Company for Pharmaceuticals and Chemical Industries, S.A.E., labeled to contain 400 mg MXF per tablet. Enrocin 10% injectable (Alexandria Company for Pharmaceuticals and Chemical Industries, Alexandria, Egypt) was labeled to contain 10% ENR and Avitryl 20% injectable (AVICO Veterinary Pharmaceuticals) was labeled to contain 200 mg ENR.

### 2.3. Preparation of Stock Standard Solutions

Stock standard solutions of GMF, MXF, and ENR (100 *μ*g mL^−1^ and 1.0 × 10^−3 ^M) were prepared by dissolving an exact weight of pure drugs in least amount of 0.1 M HCl; the mixture was warmed at 50°C in a water bath for 5.0 min, agitated by an electrical shaker for another 5.0 min, cooled to room temperature, and diluted to 100 mL with bidistilled water in a 100 mL measuring flask. The standard solutions were found stable for at least one week without alteration when kept in an amber coloured bottle and stored in a refrigerator when not in use.

### 2.4. Reagents

Bromocresol green (BCG), bromocresol purple (BCP), bromophenol blue (BPB), bromothymol blue (BTB), and methyl orange (MO) (BDH Chemicals Ltd., Poole, England) were used without further purification. Stock solutions (1.0 × 10^−3^ M) of reagents were prepared by dissolving the appropriate weight of each reagent in 10 mL of 96% ethanol and diluted to 100 mL with bidistilled water. These solutions are stable for at least one week if kept in the refrigerator.

Series of buffer solutions of KCl-HCl (pH = 1.5–4.2), NaOAc-HCl (pH = 1.99–4.92), NaOAc-AcOH (pH = 3.0–5.6), and potassium hydrogen phthalate-HCl (pH = 2.0–7.0) were prepared by following the standard methods [48].

### 2.5. General Procedures

#### 2.5.1. For GMF

Aliquots of (0.1–3.0 mL) the standard drug solution (100 *μ*g mL^−1^) were transferred to 10 mL measuring flasks and added 2.0 mL of acetate buffers of pH 3.0 and 3.5 using (BCG or BCP) and (BPB, BTB or MO), respectively and then added 2.0 mL of all reagent solutions (1.0 × 10^−3 ^M). The mixture was extracted twice with 10 mL chloroform by shaking for 2.0 min and then allowed to stand for clear separation of the two phases and the chloroform layer was passed through anhydrous sodium sulphate. The absorbance of the yellow colored complexes was measured at 420, 408, 416, 415, and 422 nm, using BCG, BCP, BPB, BTB, and MO, respectively, against corresponding reagent blank similarly prepared. All measurements were made at room temperature (25 ± 2°C). The procedures were repeated for other analyte aliquots and calibration plots were drawn to calculate the amount of drugs in unknown analyte samples.

#### 2.5.2. For MXF

Aliquots of (0.1–2.0 mL) the standard drug solution (100 *μ*g mL^−1^) were transferred to 10 mL measuring flasks and added 2.0 mL of potassium hydrogen phthalate-HCl buffer of pH 3.5 and 3.0 using BCP or MO and BPB or BTB, respectively, then added to 2.0 mL of all reagent solutions (1.0 × 10^−3 ^M). The mixture was extracted twice with 10 mL chloroform by shaking for 2.0 min and then allowed to stand for clear separation of the two phases and the chloroform layer was passed through anhydrous sodium sulphate. The absorbance of the yellow colored complexes was measured at 410, 415, 416, and 420 nm using BCP, BTB, BPB, and MO, respectively, against corresponding reagent blank similarly prepared. All measurements were made at room temperature (25 ± 2°C). The procedures were repeated for other analyte aliquots and calibration plots were drawn to calculate the amount of drugs in unknown analyte samples.

#### 2.5.3. For ENF

Aliquots of (0.2–2.4 mL) the standard drug solution (100 *μ*g mL^−1^) were transferred to 10 mL measuring flasks and added 2.0 mL of acetate buffer of pH 3.0 using BCG or BTB and then added to 2.0 mL of reagent solutions (1.0 × 10^−3 ^M). The mixture was extracted twice with 10 mL chloroform by shaking for 2.0 min, then allowed to stand for clear separation of the two phases and the chloroform layer was passed through anhydrous sodium sulphate. The absorbance of the yellow colored complexes was measured at 419 and 414 nm using BCG and BTB, respectively, against corresponding reagent blank similarly prepared. All measurements were made at room temperature (25 ± 2°C). The procedures were repeated for other analyte aliquots and calibration plots were drawn to calculate the amount of drug in unknown analyte samples.

### 2.6. Applications to Pharmaceutical Formulations

#### 2.6.1. Procedure for Tablets

The contents of ten tablets (Factive, Flobiotic, or GemiQue) labeled to contain 320 mg GMF per tablet and (Avelox or Moxiflox) labeled to contain 400 mg MXF per tablet were crushed, powdered, and weighted out and the average weight of one tablet was determined. An accurate weight equivalent to 10 mg GMF or MXF was dissolved in 20 mL of 0.5 M HCl with shaking for 5.0 min and filtered. The filtrate was diluted to 100 mL with bidistilled water in a 100 mL measuring flask to give 100 *μ*g mL^−1^ stock solution. An aliquot of the diluted drug solution was treated as described previously.

#### 2.6.2. Procedure for Injection

Accurate volumes of Enrocin 10% or Avitryl 20% of injectable quantity equivalent to 200 mg were extracted with 10 mL of 0.5 M HCl, diluted with water, and sonicated for about 5.0 min. The extracts were transferred into 100 mL volumetric flasks and then diluted to volume with bidistilled water. Aliquots of these solutions were transferred into a series of 10 mL volumetric flasks, and the analysis was completed as previously mentioned.

### 2.7. Stoichiometric Relationship

The stoichiometric ratios of the ion-associates formed between the drugs under investigation and the reagents were determined by applying the continuous variation [49] and the molar ratio [50] methods at the wavelengths of maximum absorbance. In continuous variation method, equimolar solutions were employed: 5.0 × 10^−4 ^M standard solutions of drug and 5.0 × 10^−4 ^M solutions of dye were used. A series of solutions was prepared in which the total volume of the studied drugs and the dye was kept at 2.0 mL. The drug and reagent were mixed in various complementary proportions (0 : 2, 0.2 : 1.8, 0.4 : 1.6,…,2 : 0, inclusive) and completed to volume in a 10 mL calibrated flask with the appropriate solvent for extraction following the above mentioned procedure. In the molar ratio method, the concentrations of GMF, MXF, and ENF are kept constant (1.0 mL of 5.0 × 10^−4 ^M) while that of dyes (5.0 × 10^−4 ^M) are regularly varied (0.2–2.4 mL). The absorbance of the prepared solutions optimum is measured at optimum condition at wavelength for each complex.

## 3. Results and Discussion

### 3.1. Absorption Spectra

The nitrogenous drugs are present in positively charged protonated forms and anionic dyes of sulfonephthalein group present mainly in anionic form at pH ≥ 2.5. So when treated with an acid dye at pH range 2.8–4.0 of acidic buffers solutions, a yellow ion-pair complex which is extracted with chloroform is formed. The absorption spectra of the ion-pair complexes, which were formed between GMF, MXF, or ENF and reagents, were measured in the range 350–550 nm against the blank solution. The ion-pair complexes of GMF and BCG, BCP, BPB, BTB, and MO show maximum absorbance at 420, 408, 416, 415, and 422 nm, respectively; of MXF and BCP, BTB, BPB, and MO show maximum absorbance at 410, 415, 416, and 420 nm, respectively and of ENF and BCG and BTB show maximum absorbance at 419 and 414 nm, respectively.

### 3.2. Optimum Reaction Conditions for Complex Formation

The optimization of the methods was carefully studied to achieve complete reaction formation, highest sensitivity, and maximum absorbance.

#### 3.2.1. Effects of pH on Ion-Pair Formation

**Figure 1:**
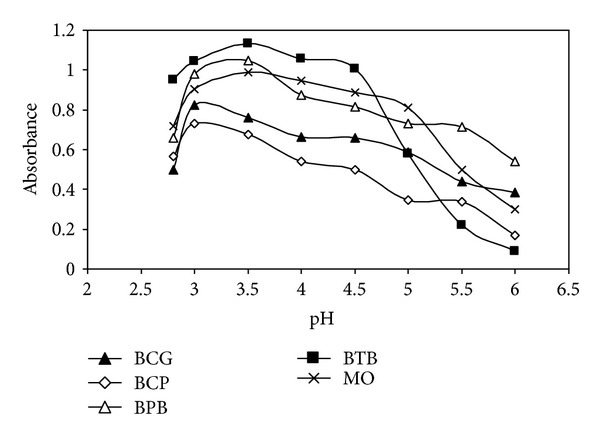
Effect of pH of acetate buffer solution on ion-pair complex formation between GMF and (1.0 × 10^−3 ^M) reagents.

The effect of pH on the drug-reagent complex was studied by extracting the colored complexes in the presence of various buffers. It was noticed that the maximum color intensity and highest absorbance value were observed in NaOAc-AcOH buffer of pH 3.0 or 3.5 using BCG or BCP and BPB, BTB, or MO, respectively, for GMF (Figure 1) and pH 3.0 using BCG or BTB for ENF. Whereas for MXF, the highest absorbance value was observed in potassium hydrogen phthalate-HCl buffer of 3.0 and 3.5 using BCP or MO and BPB or BTB, respectively, in addition to the stability of the color without affecting the absorbance at the optimum pH values. Further, 2.0 mL of the buffers solutions gave maximum absorbances and reproducible results.

#### 3.2.2. Effect of Extracting Solvents

The effect of several organic solvents, namely, chloroform, carbon tetrachloride, methanol, ethanol, acetonitrile, *n*-butanol, benzene, acetone, ethyl acetate, diethyl ether, toluene, dichloromethane, and chlorobenzene, was studied for effective extraction of the colored species from aqueous phase. Chloroform was found to be the most suitable solvent for extraction of colored ion-pair complexes for all reagents quantitatively. Experimental results indicated that double extraction with total volume 10 mL chloroform, yielding maximum absorbance intensity, stable absorbance for the studied drugs and considerably lower extraction ability for the reagent blank and the shortest time to reach the equilibrium between both phases.

#### 3.2.3. Effects of Reagents Concentration

**Figure 2:**
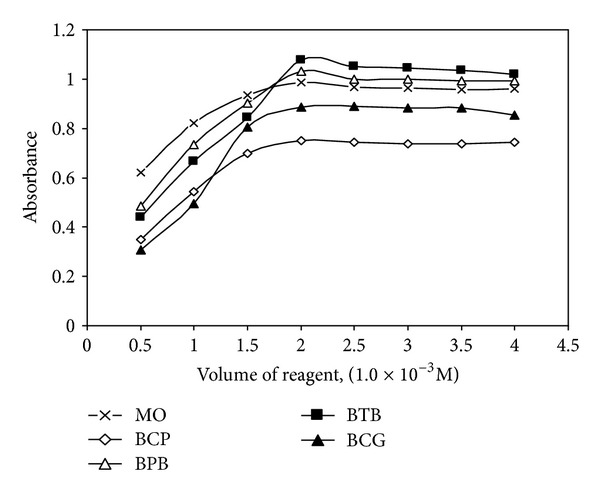
Effect of volume of (1.0 × 10^−3 ^M) reagent on the ion-pair complex formation with GMF.

The effect of the reagents was studied by measuring the absorbance of solutions containing a fixed concentration of GMF, MXF, or ENF and varied amounts of the respective reagents. Maximum color intensity of the complex was achieved with 2.0 mL of 1.0 × 10^−3^ M of all reagents solutions, although a larger volume of the reagent had no pronounced effect on the absorbance of the formed ion-pair complex (Figure 2).

#### 3.2.4. Effect of Time and Temperature

The optimum reaction time was investigated from 0.5 to 5.0 min by following the color development at ambient temperature (25 ± 2°C). Complete color intensity was attained after 2.0 min of mixing for all complexes. The effect of temperature on colored complexes was investigated by measuring the absorbance values at different temperatures. It was found that the colored complexes were stable up to 35°C. At higher temperatures, the drug concentration was found to increase due to the volatile nature of the chloroform. The absorbance remains stable for at least 12 h at room temperature for all reagents.

### 3.3. Stoichiometric Relationship

**Figure 3:**
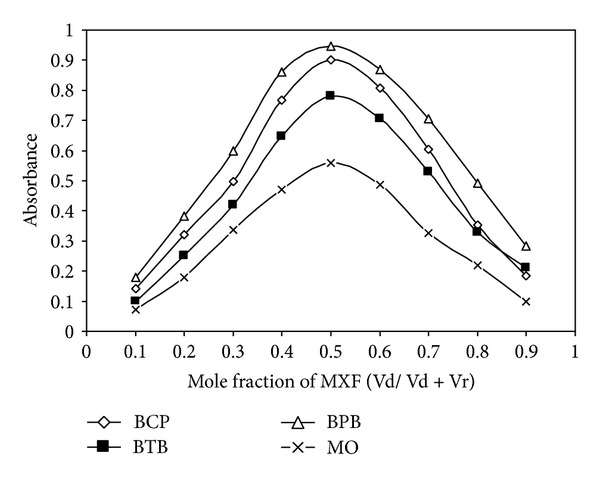
Job's method of continuous variation graph for the reaction of MXF with dyes BCP, BPB, BTB, and MO, [drug] = [dye] = 5.0 × 10^−4 ^M.

**Scheme 2:**
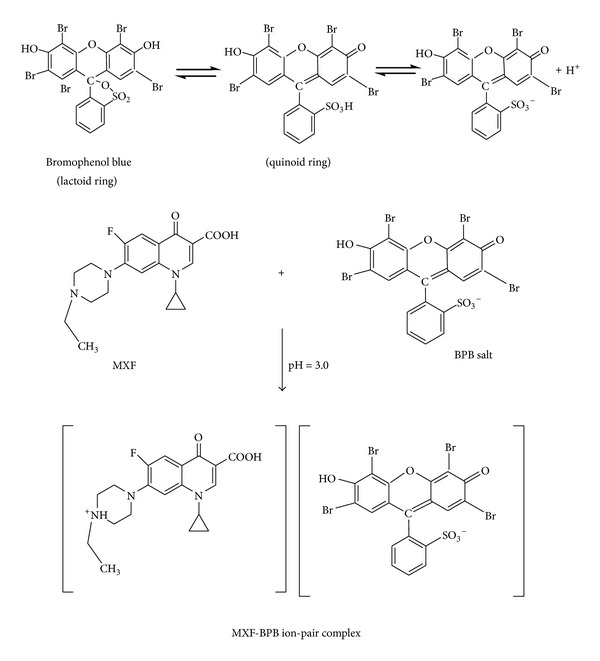
Proposed mechanism of the reaction between MXF and BPB salt.

The stoichiometric ratio between drug and dye in the ion-pair complexes was determined by the continuous variations method (Figure 3). Job's method of continuous variation of equimolar solutions was employed: a 5.0 × 10^−4^ M standard solution of drug base and 5.0 × 10^−4^ M solution of BCG, BCP, BPB, BTB, or MO, respectively, were used. A series of solutions was prepared in which the total volume of drug and reagent was kept at 2.0 mL for BCG, BCP, BPB, BTB, and MO, respectively. The absorbance was measured at the optimum wavelength. The results indicate that 1 : 1 (drug : dye) ion-pairs are formed through the electrostatic attraction between positive protonated GMF^+^, MXF^+^, or ENF^+^ and negative BCG^−^, BCP^−^, BPB^−^, BTB^−^, and MO^−^. The extraction equilibrium can be represented as follows:

(1) $$\begin{array}{c} {\text{GMF}}_{\left(\text{aq}\right)}^{+}+{\text{D}}_{\left(\text{aq}\right)}^{-}⟷\text{GM}{\text{F}}^{+}{\text{D}}_{\left(\text{aq}\right)}^{-}⟷\text{GM}{\text{F}}^{+}{\text{D}}_{\left(\text{org}\right)}^{-}, \end{array}$$

where GMF^+^ and D^−^ represent the protonated GMF and the anion of the dye, respectively, and the subscripts (aq) and (org) refer to the aqueous and organic phases, respectively (Scheme 2).

### 3.4. Conditional Stability Constants (*K*_*f*_) of Ion-Pair Complexes

The stability of the ion-pair complexes was evaluated. The formation of the ion-pair complexes was rapid and the yellow color extracts were stable at least for 12 h for drug-dye without any change in color intensity and with the maximum absorbance at room temperature. The conditional stability constants (*K*_*f*_) of the ion-pair complexes for the studied drug were calculated from the continuous variation data using the following equation [51]:

(2) $$\begin{array}{c} K_{f}=\frac{A/A_{m}}{{\left[1-A/A_{m}\right]}^{n+1}\;C_{M}{\left(n\right)}^{n}}, \end{array}$$

where *A* is the observed maximum absorbance, *A*_*m*_ is the absorbance value corresponding to intersection of the two tangents of the curve, *C*_*M*_ is the mole concentration corresponding to maximum absorbance, and *n* is the stoichiometry with which dye ion associates with drugs. The log⁡*K*_*f*_ values for drug-dye ion-pair associates were calculated in Table 1.

### 3.5. Method of Validation

#### 3.5.1. Linearity

At described experimental conditions for GMF, MXF, and ENF determination, standard calibration curves with reagents were constructed by plotting absorbance versus concentration. The statistical parameters were given in the regression equation calculated from the calibration graphs. The linearity of calibration graphs was proved by the high values of the correlation coefficient (*r*) and the small values of the *y*-intercepts of the regression equations. The apparent molar absorptivities of the resulting colored ion-pair complexes and relative standard deviation of response factors for each proposed spectrophotometric method were also calculated and recorded in Table 1. The molar absorptivity of BCP > BCG > BTB > MO > BPB ion-pair complexes for GMF, while for MXF the molar absorptivity of BCP > BTB > BPB > MO ion-pair complexes, also, the molar absorptivity of BCG > BTB ion-pair complexes for ENF.

#### 3.5.2. Sensitivity

The limits of detection (LOD) and quantitation (LOQ) for the proposed methods were calculated using the following equation [51, 52]:

(3) $$\begin{array}{c} \text{LOD}=\frac{3s}{k},\;\;\;\text{LOQ}=\frac{10s}{k}, \end{array}$$

where is the standard deviation of the response of the blank or the standard deviation of intercepts of regression lines and *k* is the sensitivity, namely, the slope of the calibration graph. In accordance with the formula, the limits of detection for GMF were found to be 0.23, 0.26, 0.52, 0.28, and 0.87 *μ*g mL^−1^ for BCG, BCP, BTB, BPB, and MO methods, respectively. Whereas, for MXF the detection limits were found to be 0.21, 0.56, 0.25, and 0.41 *μ*g mL^−1^ for BCP, BTB, BPB, and MO methods, respectively. Also, for ENF the detection limits were found to be 0.48 and 0.51 *μ*g mL^−1^ for BCG and BTB methods, respectively.

According to this equation, the limit of quantitation for GMF was found to be 0.77, 0.87, 1.73, 0.93, and 2.90 *μ*g mL^−1^ for BCG, BCP, BTB, BPB, and MO methods, respectively. Whereas, for MXF the detection limits were found to be 0.70, 1.87, 0.83, and 1.37 *μ*g mL^−1^ for BCP, BTB, BPB, and MO methods, respectively. Also, for ENF the detection limits were found to be 1.6 and 1.70 *μ*g mL^−1^ for BCG and BTB methods, respectively.

#### 3.5.3. Accuracy and Precision

Specificity of ion-pair reaction and selective determination of GMF, MXF, and ENF which were the basic nitrogenous compounds with acid dyes could be possible. Percentage relative standard deviation (RSD%) as precision and percentage relative error (RE%) as accuracy of the suggested methods were calculated. Precision was carried out by six determinations at four different concentrations in these spectrophotometric methods. The percentage relative error was calculated using the following equation:

(4) $$\begin{array}{c} \text{RE}\%=\left[\frac{\text{founded}-\text{added}}{\text{added}}\right]\times 100. \end{array}$$

The interday and intraday precision and accuracy results are shown in Tables 2, 3, and 4. These results of accuracy and precision show that the proposed methods have good repeatability and reproducibility.

#### 3.5.4. Robustness and Ruggedness

For the evaluation of the method robustness, some parameters were interchanged: pH, dye concentration, wavelength range, and shaking time. The capacity remains unaffected by small deliberate variations. Method ruggedness was expressed as RSD% of the same procedure applied by two analysts and with two different instruments on different days. The results showed no statistical differences between procedures done with different analysts and instruments suggesting that the developed methods were robust and rugged.

### 3.6. Effects of Interference

To assess the usefulness of the method, the effect of diluents, excipients, and additives which often accompany GMF, MXF, and ENF in their dosage forms (starch, lactose, glucose, sucrose, talc, sodium chloride, titanium dioxide, and magnesium stearate) was studied. The results indicated that there is no interference from excipients and additives, indicating a high selectivity for determining the studied GMF, MXF, and ENF in their dosage forms.

### 3.7. Analysis of Pharmaceutical Formulations

The proposed methods have been successfully applied to the determination of GMF, MXF, and ENF in pharmaceutical dosage forms. Six replicate determinations were made. Moreover, to check the validity of the proposed methods, dosage forms were tested for possible interference with standard addition method (Tables 5, 6, and 7). There was no significant difference between slopes of calibration curves and standard addition methods. Therefore it is concluded that the excipients in pharmaceutical dosage forms of GMF, MXF, and ENF were not found any interference in the analysis of GMF, MXF, and ENF. At 95% confidence level the calculated *t*- and *F*-values did not exceed the theoretical *F*-value indicating no significant difference between the proposed methods and the reported methods for GMF [29], MXF [40], and ENF [44] (Table 8) [52]. The results show that satisfactory recovery data were obtained and the assay results were in good agreement with the reported methods.

## 4. Conclusion

This paper describes the application of extractive ion-pair complexation reaction with acid dyes for the quantification of some fluoroquinolones antibiotics (GMF, MXF, and ENF) in pure forms and pharmaceutical formulations. Compared with the existing visible spectrophotometric methods, the proposed methods have the advantages of being relatively simple, rapid, cost-effective, free from auxiliary reagents, and more sensitive for determination of the studied drugs in pure form and pharmaceutical formulations. Moreover, the proposed methods are free from tedious experimental steps such as heating unlike the previously reported spectrophotometric methods cited earlier. The most attractive feature of these methods is their relative freedom from interference by the usual diluents and excipients in amounts far in excess of their normal occurrence in pharmaceutical formulations. The statistical parameters and the recovery data reveal high precision and accuracy of the methods besides being robust and rugged. Therefore, the validated method could be useful for routine quality control assay of the studied drugs in pure forms and pharmaceutical formulations.

## Figures and Tables

**Table 1:** Statistical analysis of calibration graphs and analytical data in the determination of the studied drugs using the proposed methods.

Parameters	GMF					MXF				ENF	
	BCG	BCP	BTB	BPB	MO	BCP	BTB	BPB	MO	BCG	BTB
Wavelengths *λ*_max⁡_ (nm)	420	408	415	416	422	410	415	416	420	419	414
pH	3.0	3.0	3.5	3.5	3.5	3.0	3.5	3.0	3.5	3.0	3.0
Beer's law limits (µg mL^−1^)	1.0–16	1.0–12	2.0–16	1.0–16	3.0–30	1.0–12	2.0–18	1.0–10	2.0–20	2.0–20	2.0–24
Molar absorptivity *ε* (L/mol^−1^ cm^−1^) × 10^4^	2.1787	3.9244	1.8904	2.4457	0.9386	3.3572	1.9365	4.1976	1.2876	1.4126	1.198
Sandell's sensitivity (ng cm^−2^)	22.3	12.4	25.7	19.9	51.7	13.0	22.6	10.4	34.0	25.4	30.0
log *K*_*f*_	5.25 ± 0.13	4.90 ± 0.10	4.95 ± 0.08	5.36 ± 0.12	4.76 ± 0.09	4.86 ± 0.07	4.98 ± 0.11	5.12 ± 0.09	5.20 ± 0.07	4.82 ± 0.12	5.14 ± 0.09
Regression equation^a^											
Intercept (*a*)	0.0016	0.0042	0.0087	0.0064	−0.0006	−0.0091	−0.0058	−0.0137	0.0299	0.0066	0.0005
Slope (*b*)	0.0447	0.0805	0.0382	0.0498	0.0196	0.0764	0.0441	0.0953	−0.0023	0.0393	0.0334
Correlation coefficient (*r*)	0.9998	0.9999	0.9993	0.9997	0.9996	0.9991	0.9997	0.9994	0.9995	0.9998	0.9995
LOD (µg mL^−1^)^b^	0.23	0.26	0.52	0.28	0.87	0.21	0.56	0.25	0.41	0.48	0.51
LOQ (µg mL^−1^)^b^	0.77	0.87	1.73	0.93	2.90	0.70	1.87	0.83	1.37	1.60	1.70
Mean ± SD	99.80 ± 1.14	99.60 ± 0.74	99.90 ± 0.90	99.75 ± 1.05	99.65 ± 0.86	99.95 ± 0.74	100.10 ± 1.07	99.60 ± 0.82	99.70 ± 0.79	100.05 ± 0.98	99.80 ± 0.87
RSD %	1.14	0.74	0.90	1.05	0.86	0.74	1.07	0.82	0.79	0.98	0.87
RE %	1.20	0.77	0.94	1.10	0.90	0.78	1.12	0.86	0.83	1.03	0.91
*t*-test^c^	0.23	0.50	0.19	0.29	0.42	0.01	0.11	0.26	0.19	0.24	0.07
*F*-test^c^	3.49	1.47	2.18	2.96	1.98	1.35	1.54	1.10	1.19	1.24	1.02

^a^ *A* = *a* + *bC*, where *C* is the concentration in µg mL^−1^ and *y* is the absorbance units.

^b^LOD: limit of detection; LOQ: limit of quantification; *ε*: molar absorptivity.

^c^The theoretical values of *t* and *F* at *P* = 0.05 are 2.571 and 5.05, respectively.

**Table 2:** Intraday and interday precision and accuracy data for GMF obtained by the proposed methods.

Method	Added (*μ*g mL^−1^)	Intraday				Interday			
		Recovery %	Precision RSD %^a^	Accuracy RE %	Confidence limit^b^	Recovery %	Precision RSD %^a^	Accuracy RE %	Confidence limit^b^
BCG	5.0	99.30	0.53	−0.80	4.996 ± 0.026	100.60	0.43	0.60	5.03 ± 0.022
	10	99.50	0.65	−0.50	9.950 ± 0.065	99.30	0.59	−0.70	9.930 ± 0.059
	15	99.10	0.82	−0.90	14.865 ± 0.122	99.90	0.83	−0.10	14.985 ± 0.124
BCP	4.0	100.60	0.47	0.60	4.024 ± 0.019	99.30	0.51	−0.70	3.988 ± 0.02
	8.0	99.20	0.72	−0.80	7.936 ± 0.057	99.70	0.70	−0.30	7.976 ± 0.056
	12	100.20	1.17	0.20	12.024 ± 0.141	100.30	1.10	0.30	12.036 ± 0.132
BPB	5.0	100.10	0.49	0.10	5.005 ± 0.025	99.60	0.62	−0.40	4.98 ± 0.031
	10	99.80	0.79	−0.20	9.98 ± 0.079	99.40	0.87	−0.60	9.94 ± 0.086
	15	99.70	1.01	−0.30	14.955 ± 0.151	100.20	1.24	0.20	15.03 ± 0.23
BTB	5.0	99.60	0.65	−0.40	4.98 ± 0.032	99.70	0.54	−0.30	4.985 ± 0.027
	10	100.10	0.88	0.10	10.01 ± 0.088	99.30	0.75	−0.70	9.93 ± 0.074
	15	100.40	0.93	0.40	15.06 ± 0.140	100.20	1.08	0.20	15.03 ± 0.162
MO	10	99.60	0.44	−0.40	9.96 ± 0.044	99.50	0.52	−0.50	9.95 ± 0.052
	20	99.80	0.68	−0.20	19.96 ± 0.136	99.70	0.83	−0.30	19.94 ± 0.166
	30	100.30	0.98	0.30	30.09 ± 0.295	100.20	1.20	0.20	30.06 ± 0.361

^a^Mean of six determinations; RSD %: percentage relative standard deviation; RE %: percentage relative error.

^b^Confidence limit at 95% confidence level and five degrees of freedom (*t* = 2.571).

**Table 3:** Intraday and interday precision and accuracy data for MXF obtained by the proposed methods.

Method	Added (*μ*g mL^−1^)	Intraday				Interday			
		Recovery %	Precision RSD %^a^	Accuracy RE %	Confidence limit^b^	Recovery %	Precision RSD %^a^	Accuracy RE %	Confidence limit^b^
BCP	2.0	99.50	0.36	−0.50	1.99 ± 0.007	99.00	0.38	−1.00	1.98 ± 0.008
	6.0	100.20	0.57	0.20	6.012 ± 0.034	99.60	0.70	−0.40	5.976 ± 0.042
	10	100.50	0.81	0.50	10.05 ± 0.081	99.80	0.92	−0.20	9.98 ± 0.092
BPB	5.0	99.20	0.42	−0.80	4.96 ± 0.021	100.30	0.50	0.30	5.015 ± 0.025
	10	99.50	0.76	−0.50	9.95 ± 0.076	99.20	0.79	−0.80	9.92 ± 0.078
	15	99.70	0.90	−0.30	14.955 ± 0.135	99.60	1.16	−0.40	14.94 ± 0.173
BTB	2.0	99.40	0.50	−0.60	1.988 ± 0.01	100.10	0.58	0.10	2.002 ± 0.012
	6.0	99.20	0.79	−0.80	5.952 ± 0.047	99.70	0.76	−0.30	5.982 ± 0.045
	10	99.50	1.05	−0.50	9.95 ± 0.104	99.30	1.07	−0.70	9.93 ± 0.106
MO	5.0	100.30	0.42	0.30	5.015 ± 0.021	100.40	0.47	0.40	5.02 ± 0.024
	10	100.60	0.85	0.60	10.06 ± 0.086	99.80	0.83	−0.20	9.98 ± 0.083
	15	99.90	1.19	−0.10	14.985 ± 0.178	99.20	1.23	−0.80	14.88 ± 0.183

^a^Mean of six determinations; RSD %: percentage relative standard deviation; RE %: percentage relative error.

^b^Confidence limit at 95% confidence level and five degrees of freedom (*t* = 2.571).

**Table 4:** Intraday and interday precision and accuracy data for ENF obtained by the proposed methods.

Method	Added (*μ*g mL^−1^)	Intraday				Interday			
		Recovery %	Precision RSD %^a^	Accuracy RE %	Confidence limit^b^	Recovery %	Precision RSD %^a^	Accuracy RE %	Confidence limit^b^
BCG	5.0	99.10	0.42	−0.90	4.955 ± 0.021	99.60	0.51	−0.40	4.98 ± 0.025
	10	100.20	0.78	0.20	10.02 ± 0.078	99.80	0.75	0.20	9.98 ± 0.075
	15	99.40	1.16	−0.60	14.91 ± 0.173	99.20	1.02	−0.80	14.88 ± 0.152
BTB	5.0	99.30	0.53	−0.70	4.965 ± 0.026	100.60	0.40	0.60	5.03 ± 0.020
	10	99.50	0.90	−0.50	9.95 ± 0.09	99.40	0.68	−0.60	9.94 ± 0.068
	20	100.50	1.15	0.50	20.10 ± 0.231	99.60	0.96	−0.40	19.92 ± 0.191

^a^Mean of six determinations; RSD%: percentage relative standard deviation; RE%: percentage relative error.

^b^Confidence limit at 95% confidence level and five degrees of freedom (*t* = 2.571).

**Table 5:** Determination of GMF in its pharmaceutical dosage forms applying the standard addition technique.

Reagent	Taken (*μ*g mL^−1^)	Pure drug added (*μ*g mL^−1^)	Factive tablets		Flobiotic tablet		GemiQue tablets	
			Total found (*μ*g mL^−1^)	Recovery %^a^ ± SD	Total found (*μ*g mL^−1^)	Recovery % ± SD	Total found (*μ*g mL^−1^)	Recovery % ± SD
BCG	2.0	4.0	5.96	99.30 ± 0.78	5.98	99.60 ± 0.67	6.02	100.30 ± 0.51
		8.0	9.91	99.10 ± 1.02	10.02	100.20 ± 0.82	9.92	99.20 ± 0.70
		12	13.97	99.80 ± 1.24	13.96	99.70 ± 1.09	13.99	99.90 ± 0.94
BCP	2.0	2.0	3.996	99.90 ± 0.66	3.96	99.10 ± 0.42	3.97	99.30 ± 0.38
		6.0	8.02	100.20 ± 0.90	7.98	99.80 ± 0.69	7.99	99.90 ± 0.84
		10	11.92	99.30 ± 0.95	12.05	100.40 ± 1.03	11.95	99.60 ± 1.17
BPB	2.0	4.0	5.97	99.50 ± 0.57	5.99	99.80 ± 0.36	6.01	100.15 ± 0.76
		8.0	9.97	99.70 ± 0.76	9.96	99.60 ± 0.49	9.97	99.70 ± 0.96
		12	13.87	99.10 ± 1.14	13.97	99.90 ± 0.78	13.89	99.20 ± 1.21
BTB	2.0	4.0	6.03	100.50 ± 0.48	6.01	100.10 ± 0.53	6.01	100.10 ± 0.54
		8.0	9.90	99.00 ± 0.72	9.96	99.60 ± 0.85	9.96	99.60 ± 1.03
		12	13.93	99.50 ± 0.97	13.96	99.70 ± 1.31	14.04	100.30 ± 1.15
MO	5.0	5.0	9.97	99.70 ± 0.32	9.91	99.10 ± 0.70	9.99	99.90 ± 0.65
		10	14.94	99.60 ± 0.56	15.06	100.40 ± 1.05	14.97	99.80 ± 0.85
		20	24.95	99.80 ± 0.90	24.88	99.50 ± 1.25	24.90	99.60 ± 1.10

^a^Average of six determinations.

**Table 6:** Determination of MXF in its pharmaceutical dosage forms applying the standard addition technique.

Reagent	Taken (*μ*g mL^−1^)	Pure drug added (*μ*g mL^−1^)	Avelox tablets		Moxiflox tablets		Moxifloxacin tablets	
			Total found (*μ*g mL^−1^)	Recovery %^a^ ± SD	Total found (*μ*g mL^−1^)	Recovery % ± SD	Total found (*μ*g mL^−1^)	Recovery % ± SD
BCP	2.0	2.0	3.98	99.40 ± 0.56	3.96	99.10 ± 0.62	3.98	99.60 ± 0.48
		6.0	7.98	99.70 ± 0.83	8.06	100.70 ± 0.89	7.98	99.80 ± 1.10
		10	11.89	99.10 ± 1.20	12.02	100.20 ± 1.26	11.92	99.30 ± 1.17
BPB	2.0	4.0	5.98	99.60 ± 0.61	5.97	99.50 ± 0.35	5.96	99.30 ± 0.36
		8.0	9.99	99.90 ± 0.84	9.96	99.60 ± 0.60	9.99	99.90 ± 0.63
		12	14.07	100.50 ± 1.14	13.99	99.90 ± 0.85	14.08	100.60 ± 1.36
BTB	2.0	2.0	3.98	99.50 ± 0.48	3.97	99.20 ± 0.33	4.01	100.20 ± 0.61
		4.0	5.95	99.20 ± 0.92	5.99	99.80 ± 0.65	5.96	99.30 ± 0.84
		8.0	10.04	100.40 ± 1.17	10.01	100.10 ± 0.93	9.95	99.50 ± 1.07
MO	5.0	5.0	9.91	99.10 ± 0.42	9.94	99.40 ± 0.37	9.95	99.50 ± 0.45
		10	15.08	100.50 ± 0.76	15.03	100.20 ± 0.75	14.97	99.80 ± 0.85
		15	19.90	99.50 ± 1.16	19.92	99.60 ± 1.28	19.83	99.15 ± 0.90

^a^Average of six determinations.

**Table 7:** Determination of ENF in its pharmaceutical dosage forms applying the standard addition technique.

Reagent	Taken (*μ*g mL^−1^)	Pure drug added (*μ*g mL^−1^)	Enrocin 10% injectable		Avitryl 20% injectable	
			Total found (*μ*g mL^−1^)	Recovery %^a^ ± SD	Total found (*μ*g mL^−1^)	Recovery % ± SD
BCG	5.0	5.0	9.92	99.20 ± 0.48	9.94	99.40 ± 0.46
		10	14.94	99.60 ± 0.66	15.20	100.10 ± 0.72
		15	19.98	99.90 ± 0.82	19.90	99.50 ± 1.05
BBTB	5.0	5.0	10.05	100.50 ± 0.56	9.97	99.70 ± 0.52
		10	14.91	99.40 ± 0.83	15.05	100.30 ± 0.69
		15	19.84	99.20 ± 1.20	19.82	99.10 ± 0.95

^a^Average of six determinations.

**Table 8:** Application of the proposed methods for the determination of GMF, MXF, and ENF in their pharmaceutical preparations.

Samples	Reported methods^c^	Proposed methods				
		BCG	BCP	BPB	BTB	MO
Factive tablets						
X ± SD^a^	100.08 ± 0.56	99.90 ± 0.62	100.15 ± 0.74	99.75 ± 0.53	99.80 ± 0.71	100.20 ± 0.59
*t*-value^b^		0.20	0.07	0.39	0.28	0.13
*F*-value^b^		1.23	1.75	1.12	1.61	1.11
Flobiotic tablets						
X ± SD^a^	99.94 ± 0.68	99.68 ± 0.80	99.79 ± 0.57	99.90 ± 0.73	100.10 ± 0.84	100.20 ± 0.77
*t*-value^b^		0.23	0.15	0.04	0.14	0.23
*F*-value^b^		1.38	1.42	1.15	1.53	1.67
GemiQue tablets						
X ± SD^a^	99.85 ± 0.49	99.70 ± 0.60	100.05 ± 0.57	99.60 ± 0.38	99.96 ± 0.55	99.55 ± 0.63
*t*-value^b^		0.18	0.24	0.37	0.14	0.34
*F*-value^b^		1.50	1.35	1.66	1.26	1.65
Avelox tablets						
X ± SD^a^	99.03 ± 0.97		99.60 ± 0.74	99.35 ± 0.96	99.10 ± 1.20	99.50 ± 0.82
*t*-value^b^			0.47	0.21	0.04	0.34
*F*-value^b^			1.72	1.02	1.53	1.40
Moxiflox tablets						
X ± SD^a^	99.34 ± 0.34		99.15 ± 0.52	99.50 ± 0.46	99.62 ± 0.43	99.55 ± 0.60
*t*-value^b^			0.28	0.26	0.47	0.28
*F*-value^b^			2.34	1.83	1.60	3.11
Moxifloxacin tablets						
X ± SD^a^	99.94 ± 0.92		99.70 ± 1.05	99.85 ± 0.80	100.15 ± 0.98	99.90 ± 0.84
*t*-value^b^			0.16	0.07	0.14	0.03
*F*-value^b^			1.30	1.32	1.13	1.20
Enrocxin 10% injectable						
X ± SD^a^	99.85 ± 0.43	99.70 ± 0.68			100.10 ± 0.32	
*t*-value^b^		0.17			0.43	
*F*-value^b^		2.50			1.81	
Avitryl 20% injectable						
X ± SD^a^	99.78 ± 0.64	99.50 ± 0.48			99.46 ± 0.47	
*t*-value^b^		0.32			0.37	
*F*-value^b^		1.78			1.85	

^a^Average of six determinations.

^b^Theoretical values for *t*- and *F*-values at five degrees of freedom and 95% confidence limit are *t* = 2.57 and *F* = 5.05.

^c^Reported spectrophotometric methods for GMF [29], MXF [40], and ENF [44].
